# The Aqueous Extract of Radio-Resistant *Deinococcus actinosclerus *BM2T Suppresses Lipopolysaccharide-Mediated Inflammation in RAW264.7 Cells

**DOI:** 10.4014/jmb.1911.11003

**Published:** 2019-12-15

**Authors:** Myung Kyum Kim, Seon-A Jang, Seung Namkoong, Jin Woo Lee, Yuna Park, Sung Hyeok Kim, Sung Ryul Lee, Eun-Hwa Sohn

**Affiliations:** 1 Department of Bio & Environmental Technology, College of Natural Science, Seoul Women’s University, Seoul 01797, Republic of Korea; 2 Herbal Medicine Research Division, Korea Institute of Oriental Medicine, Daejeon 34054, Republic of Korea; 3 College of Health Sciences, Kangwon National University, Samcheok 25949, Republic of Korea; 4 Department of Convergence Biomedical Science, Cardiovascular and Metabolic Disease Center, College of Medicine, Inje University, Busan 47392, Republic of Korea

**Keywords:** *Deinococcus actinosclerus* BM2^T^, lipopolysaccharide, cytokines, anti-inflammatory, macrophages

## Abstract

*Deinococcus actinosclerus* BM2^T^ (GenBank: KT448814) is a radio-resistant bacterium that is newly isolated from the soil of a rocky hillside in Seoul. As an extremophile, *D. actinosclerus* BM2^T^ may possess anti-inflammatory properties that may be beneficial to human health. In this study, we evaluated the anti-inflammatory effects of BM2U, an aqueous extract of *D. actinosclerus* BM2^T^, on lipopolysaccharide (LPS)-mediated inflammatory responses in RAW264.7 macrophage cells. BM2U showed antioxidant capacity, as determined by the DPPH radical scavenging (IC_50_ = 349.3 μg/ml) and ORAC (IC_50_ = 50.24 μg/ml) assays. At 20 μg/ml, BM2U induced a significant increase in heme oxygenase-1 (HO-1) expression (*p* < 0.05). BM2U treatment (0.2-20 μg/ml) significantly suppressed LPS-induced increase in the mRNA expression of proinflammatory cytokines tumor necrosis factor-α (TNF-α), interleukin (IL)-1β, and IL-6 (*p* < 0.05). BM2U treatment also suppressed the expression of inducible nitric oxide synthase (iNOS) and cyclooxygenase-2 (COX-2), which are involved in the production of inflammatory mediators. BM2U treatment also inhibited the activation of nuclear factor-κB (NF-κB) and mitogen-activated protein kinases (MAPKs): JNK, ERK, and p-38 (*p* < 0.05). Collectively, BM2U exhibited anti-inflammatory potential that can be exploited in attenuating inflammatory responses.

## Introduction

Inﬂammation occurs in response to any type of bodily injury, including infection, and is marked by neutrophil and macrophage recruitment leading to the production of proinflammatory cytokines and chemokines [1, 2]. Inflammation is controlled by complex regulatory mechanisms [3]. However, if poorly resolved, it may result in chronic inflammation, dubbed as the major organ dysfunction associated with fibrosis [4, 5]. Macrophages play an important role in resistance against bacterial pathogens, tissue remodeling, repair, and inflammation resolution. Furthermore, resting macrophages (M0) can be polarized to M1 and M2 in response to different stimuli, such as cytokines, microbes, and other modulators [6]. M1 macrophages (killer type cells) are activated by interferon-γ and/or lipopolysaccharides (LPS), gram-negative bacterial endotoxins that can stimulate the secretion of a variety of proinflammatory cytokines and enzymes, including inducible nitric oxide synthase (iNOS) and cyclooxygenase-2 (COX-2) [7]. M1 macrophages then secrete proinflammatory cytokines, such as tumor necrosis factor-α (TNF-α), interleukin (IL)-1β, IL-6, and IL-12. M1 macrophages are also involved in the generation of reactive oxygen species (ROS), such as nitric oxide via iNOS [8]. In contrast, M2 macrophages (repair type cells), which are induced by IL-4, IL-13, or glucocorticoid exposure, are involved in inflammation resolution *via* the secretion of anti-inflammatory cytokines (IL-10 and transforming growth factor-β) and arginase-1, which is involved in ornithine production [8].

Extremophiles, microorganisms with the ability to survive in extreme environmental conditions, may produce radiation-responsive metabolites, pigments, and enzymes for their survival [9]. Their biological ability to adapt to harsh environmental conditions has been extensively exploited for the development of therapeutic drugs, especially anti-cancer drugs, antibiotics, and agricultural products of commercial significance [9-11]. It is speculated that radio-resistant genus *Deinococcus* contains special proteins (such as ROS-scavenging enzymes, carotenoids, and manganese complexes) or mechanisms which provide protection against radiation-induced cellular damage. Among them, mycosporine-like amino acids [12], scytonemin [13], bacterioruberin, and pannarin, which show antioxidant activity, have been used in the development of cosmetics, such as sunscreens [9]. Previously, we isolated the radiation-resistant *Deinococcus actinosclerus* BM2^T^ (GenBank: KT448814) from the soil of a rocky hillside in Seoul [14]. The BM2^T^ strain is gram-positive, catalase- and oxidase-positive, and of the coccus- or rod-shaped bacterial strain. Menaquinone-8, a kind of vitamin K2, is the predominant respiratory quinone found in the BM2^T^ strain [14]. To find a valuable bioactive compound from a bacterial source, we hypothesized that radiation-resistant genus *Deinococcus* may possess anti-inflammatory properties based on their excellent viability under extreme conditions [14, 15]. Using BM2U, an aqueous extract of *D. actinosclerus* BM2^T^, we investigated its anti-inflammatory effects on LPS-challenged RAW264.7 macrophages. First, the antioxidant capacities of BM2U were determined through α, α-diphenyl-β-picrylhydrazyl (DPPH) radical scavenging activity and oxygen radical absorbance capacity (ORAC) assay. Next, the suppressive effects of BM2U on the LPS-mediated increase in TNF-α, IL-1β, and IL-6 expression levels in RAW264.7 macrophages were determined using quantitative RT-PCR (qRT-PCR). The activation of NF-κB and mitogen-activated protein kinases (MAPKs: ERK, JNK, and p38 MAPK) were determined through immunoblot analysis. LPS-insult associated increase in iNOS and COX-2 protein expression levels were determined in the presence of BM2U. Finally, the induction of heme oxygenase-1 (HO-1), which plays a pivotal role in inhibiting the progression of inflammatory response [16, 17], was determined through immunoblot analysis.

## Materials and Methods

### Chemicals and Reagents

Dulbecco’s modified Eagle media (DMEM), fetal bovine serum (FBS), trypsin-EDTA, penicillin, and streptomycin were purchased from WelGene (Korea). Lipopolysaccharides from *Escherichia coli* O55:B5, α, α-diphenyl-β-picrylhydrazyl (DPPH), β-phycoerythrin (β-PE), 2,2'-azobis (2-methylpropionamidine) dihydrochloride (AAPH), and methylthiazolyldiphenyl-tetrazolium bromide (MTT) were purchased from Sigma-Aldrich (USA). Primary antibodies against the total and phosphorylated forms of ERK1/2, JNK, p38 MAPK, the phosphorylated form of p65, HO-1, β-actin, and secondary antibodies were obtained from Cell signaling Technologies (USA). The primary antibody against IκBα was purchased from Santa Cruz Biotechnology (Santa Cruz, USA). Unless indicated otherwise, all other chemicals were obtained from Sigma-Aldrich .

### BM2U Extract Preparation

*Deinococcus actinosclerus* BM2^T^ (GenBank: KT448814) was isolated and identified from a soil sample collected from the rocky hillside of a mountain located near Seoul Women’s University, after irradiating 3 kGy using a Co^60^ gamma irradiator (point source AECL, IR-79) [14]. BM2^T^ strains were cultured with continuous shaking at 30°C in R2A medium for approximately 3 days till the late logarithmic growth phase. The cultured BM2^T^ strains were precipitated using a centrifuge and dispersed by adding distilled water. The same procedure was repeated twice to collect pure BM2^T^ strains, hence, only BM2^T^ strains were collected. Distilled water at twice the BM2^T^ strain volume, was added to the collected BM2^T^ strains and cultured with continuous shaking at 100°C for 15 min, followed by centrifugation to separate the supernatant and precipitate, and lyophilized. BM2U, being the aqueous extract of BM2^T^, only contained water-soluble components. BM2U was then diluted either in the PBS buffer or culture medium and used for the experiments. To test for possible endotoxin contamination, QCL-1000 Chromogenic Limulus Amebocyte Lysate end-point assay from Lonza (USA) was performed, as previously described [18]. The endotoxin level of a 10 mg/ml BM2U preparation was <0.1 EU/ml.

### DPPH Radical Scavenging Activity

Different concentrations of BM2U were adjusted to 100 μl with reaction mixture, and then reacted with 100 μl of 0.4 mM 2,2-diphenyl-1-picrylhydrazyl (DPPH) solution in 99% EtOH. After vigorous shaking, reaction mixtures were allowed to reach a steady state at room temperature for 30 min. DPPH decolorization was determined by measuring the absorbance at 517 nm, using a microplate reader VICTOR X3 (PerkinElmer, USA). The half-maximal inhibitory concentrations (IC_50_) of DPPH radical formation were calculated from the graph by plotting the inhibition percentages against the tested BM2U concentrations.

### Oxygen Radical Absorbance capacity (ORAC) Assay

The oxygen radical absorbance capacity (ORAC) assay was performed as previously described [19]. Trolox is a water-soluble analog of vitamin E, commonly used as the standard. Briefly, 20 μl of BM2U or Trolox at equal concentrations was incubated with 10 μM of β-PE and 50 mM of AAPH in a total volume of 200 μl. β-PE and AAPH were used as the fluorescent probe and a peroxy radical generator, respectively. The determination of a decreasing amount in fluorescence was followed at 2 min intervals for 60 min at 37°C. All ORAC analyses were performed on a microplate reader VICTOR X3 at 37°C, with an excitation wavelength of 530 nm and an emission wavelength of 590 nm. After obtaining the area under the curve (AUC) for each sample and standard, the BM2U ORAC values at different concentrations were expressed as a μM of Trolox Equivalents (TE), by comparing to the standard curve.

### Cell Culture

RAW264.7 (murine macrophage) cells were purchased from the American Type Culture Collection; ATCC (USA), and maintained in DMEM medium containing 10% FBS and 1% penicillin/streptomycin (10,000 U pen/ml and 10,000 μg strep/ml) in a humidified atmosphere of 5% CO_2_ at 37°C.

### MTT Assay

RAW264.7 cells were attached to a 96-well microplate and treated with different doses (6.25, 12.5, 25, 50, and 100 μg/ml) of BM2U for 24 h. After incubation with MTT (150 μg/ml) for 4 h, the formazan crystals formed were dissolved in DMSO and the absorbance was measured at 540 nm using a microplate reader.

### Quantitative Real-Time Polymerase Chain Reaction (qRT-PCR)

The mRNA expression levels of IL-1β, IL-6, and TNF-α were determined using reverse transcription-polymerase chain reaction (RT-PCR). RAW264.7 cells were attached to a 6-well microplate and treated with different doses (0.2, 2, and 20 μg/ml) of BM2U, with or without LPS (1 μg/ml), for 16 h. Total RNA was extracted from RAW264.7 macrophages using the TRIzol reagent from Invitrogen (USA), and processed using a cDNA Synthesis kit from TAKARA (Japan). SYBR master mix kit from TAKARA was used for RT-PCR, and cDNA was amplified using specific primers (Table 1). Quantitative real-time RT-PCR reactions were performed on a Light Cycler 96 Instrument from Roche (Basel, Switzerland). Relative quantitative evaluation of each gene was performed by the comparative cycle threshold method [20].

### Immunoblot Analyses

RAW264.7 cells were attached to a 6-well microplate and treated with different doses (0.2, 2, and 20 μg/ml) of BM2U, with or without LPS (1 μg/ml), for 15 min (p-p65, IκBα, and MAPKs) or for 24 h (COX-2, iNOS, and HO-1). The cells were then harvested, washed with cold PBS, and lysed with a homogenized PRO-PREP™ Protein Extraction Buffer from Intron Biotechnology (Korea), for 1 h on ice. After centrifugation at 15,000 ×*g* for 30 min at 4°C, the lysates were collected and the protein concentration determined using a protein assay kit from Bio-Rad Laboratories (USA), with bovine serum albumin (BSA) as the standard. Equal amounts of protein were subjected to 10% SDS-polyacrylamide gel electrophoresis (Bio-Rad Laboratories) and electrophoretically transferred to a nitrocellulose membrane. The membrane was blocked with 5% non-fat skimmed milk and incubated with appropriate primary antibodies. The blots were developed using enhanced horseradish peroxidase-conjugated anti-rabbit secondary antibodies. Blots were re-probed with an anti-β-actin antibody as a control for protein loading. Bands were visualized with the EZ-Western Lumi Pico reagent from Daeil Laboratory Service (Korea), and subjected to densitometric analyses.

### Statistical Analyses

Data are presented as mean ± standard error of the mean (SEM), from at least three independent experiments performed in triplicates. Statistically significant differences between control and experimental values were calculated with analysis of variance (ANOVA) followed by Tukey’s test, using GraphPad Prism 5 from GraphPad Software Inc. (USA). A *p*-value <0.05 was considered statistically significant.

## Results

### Antioxidant Capacities of BM2U

**Fig. 1:**
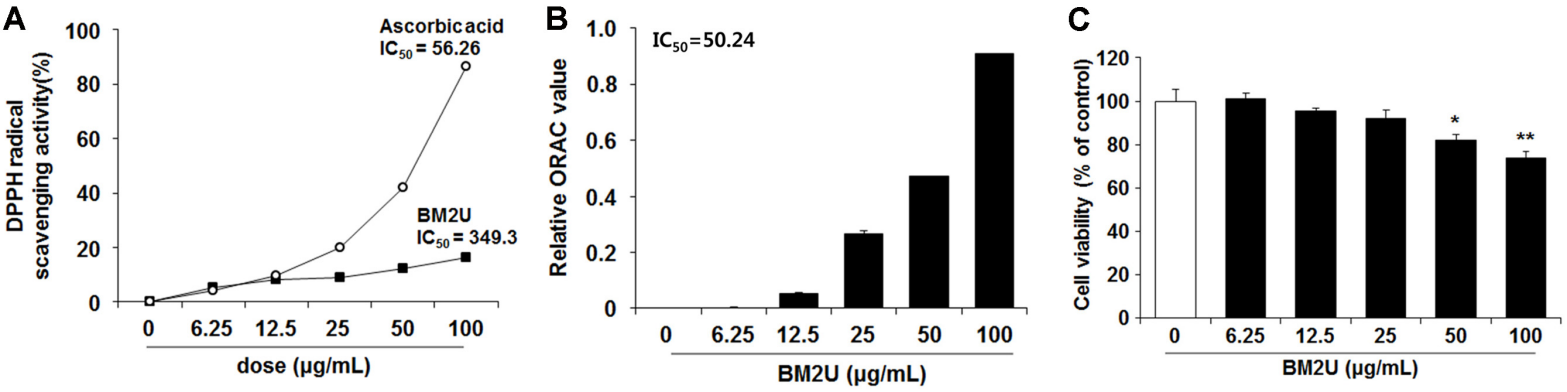
Effect of BM2U on DPPH radical scavenging activity, ORAC, and cell cytotoxicity. The antioxidant capacities of BM2U were determined through the DPPH radical scavenging activity (A) and ORAC (B) assays. RAW264.7 macrophages were incubated with different doses of BM2U for 24 h, and cell viability was determined using the MTT assay (C). *p < 0.05 or **p < 0.01 vs. the untreated control. Values represent mean ± SEM (*n* = 4, per group). BM2U; an aqueous extract of *D. actinosclerus* BM2^T^ (GenBank: KT448814).

ROS suppression via quenching free oxygen radicals is critically important in attenuating inflammatory responses. Thus, we determined the antioxidant capacities of BM2U (6.25-100 μg/ml), via the DPPH radical scavenging activity (Fig. 1A) and peroxy-radical scavenging ORAC assay (Fig. 1B). The IC_50_ values of BM2U on DPPH radical scavenging and ORAC were 349.3 μg/ml and 50.24 μg/ml, respectively. These results indicate that BM2U possesses radical scavenging capacity, favorable for direct or indirect resolution of ROS-mediated inflammation.

### BM2U Cytotoxicity

BM2U cytotoxicity in RAW264.7 macrophage cells was evaluated at 24 h, by MTT assay. There was no significant toxicity up to 25 μg/ml, but cytotoxicity was observed above 50 μg/ml of BM2U (Fig. 1C). Thus, the treatment doses of BM2U for the cells were set at 0.2, 2, and 20 μg/ml, to avoid toxicity.

### Suppressive Effect of BM2U on LPS-Mediated TNF-α, IL-1β, and IL-6 Expression

**Fig. 2:**
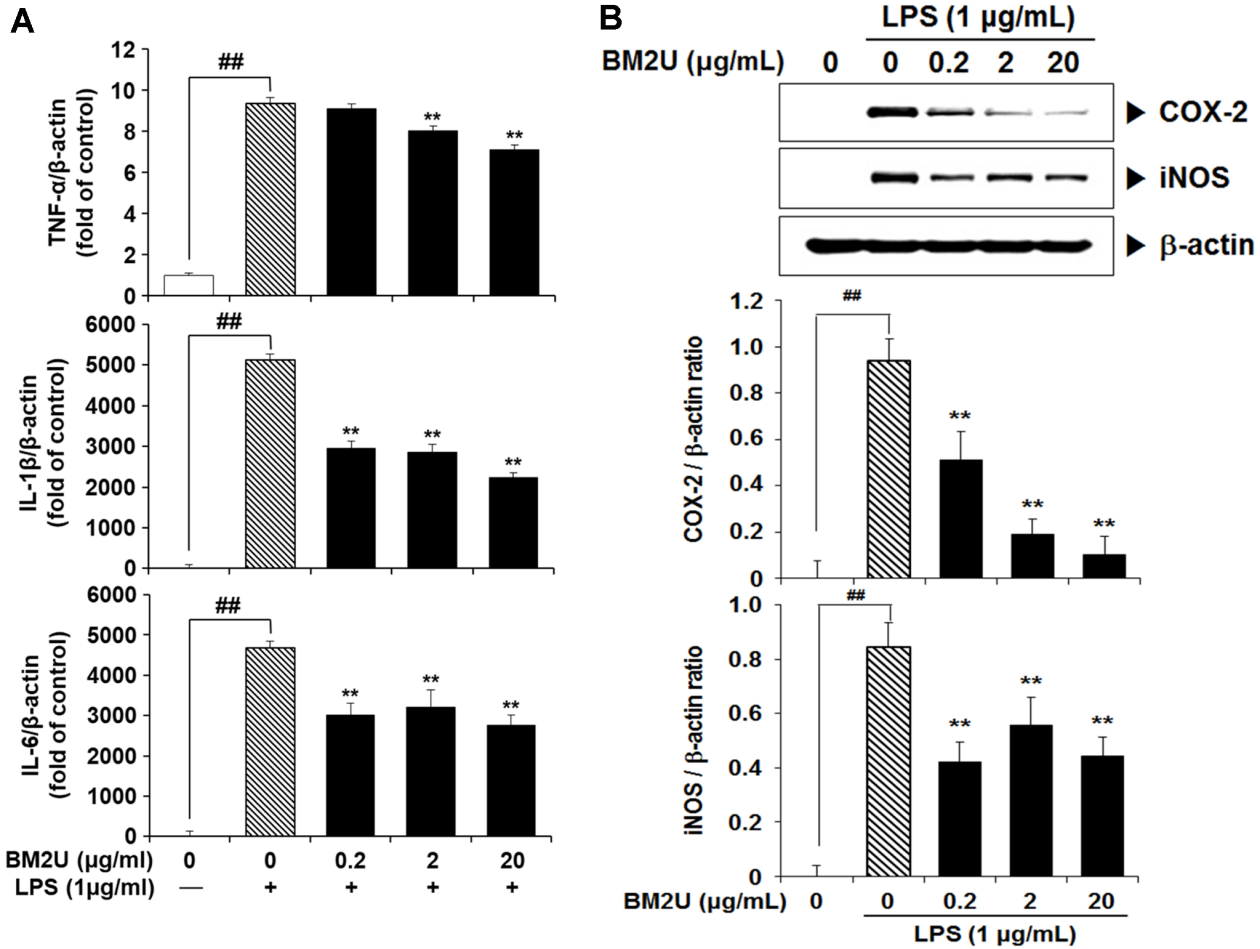
Effect of BM2U on LPS-mediated TNF-α, IL-1β, and IL-6 mRNA expression, and COX-2 and iNOS expression. RAW264.7 macrophage cells were challenged with LPS (1 μg/ml) in the presence or absence of BM2U (0.2-20 μg/ml) for 16 h, and the mRNA levels of TNF-α, IL-1β, and IL-6 were quantified using qRT-PCR. Values represent the relative ratio to β-actin as mean ± SEM (*n* = 4, per group). COX-2 and iNOS expression levels at 24 h were quantified by immunoblotting. ##*p <* 0.01 vs. the untreated control; **p <0.01 vs. the LPS-only group. BM2U, an aqueous extract of *D. actinosclerus* BM2^T^ (GenBank: KT448814).

LPS-induced monocyte and macrophage stimulation induces many genes that express proinflammatory cytokines such as TNF-α, IL-1β, and IL-6 [7, 21]. As depicted in Fig. 2A, the LPS-challenge on RAW264.7 macrophages led to significant induction of TNF-α, IL-1β, and IL-6 mRNA (*p* < 0.05). However, these increased mRNA levels were significantly inhibited in the presence of BM2U (*p* < 0.05). Although dose-dependence was not observed, BM2U-induced suppression of IL-1β and IL-6 mRNA levels was effective even at 0.2 μg/ml BM2U. These results suggest that BM2U can suppress LPS-mediated production of proinflammatory cytokines.

### Suppressive Effect of BM2U on COX-2 and iNOS Expression

NO and prostaglandin E2 (PGE2) are important inflammatory mediators produced by iNOS and COX-2, respectively. No significant COX-2 and iNOS expression was observed at baseline. However, upon LPS-induced stimulation of RAW264.7 macrophage cells, iNOS and COX-2 expression were significantly upregulated, as shown in Fig. 2B (*p <* 0.05). BM2U treatment significantly dose-dependently inhibited LPS-mediated COX-2 induction (*p* < 0.05). However, significant suppression of LPS-induced iNOS activation was not dose-dependent (*p* < 0.05). These results imply that BM2U treatment reduces LPS-induced inflammation associated with the production of PGE2 and NO, via inhibiting COX-2 and iNOS expression, respectively.

### Suppressive Effect of BM2U on NF-κB Activation

**Fig. 3:**
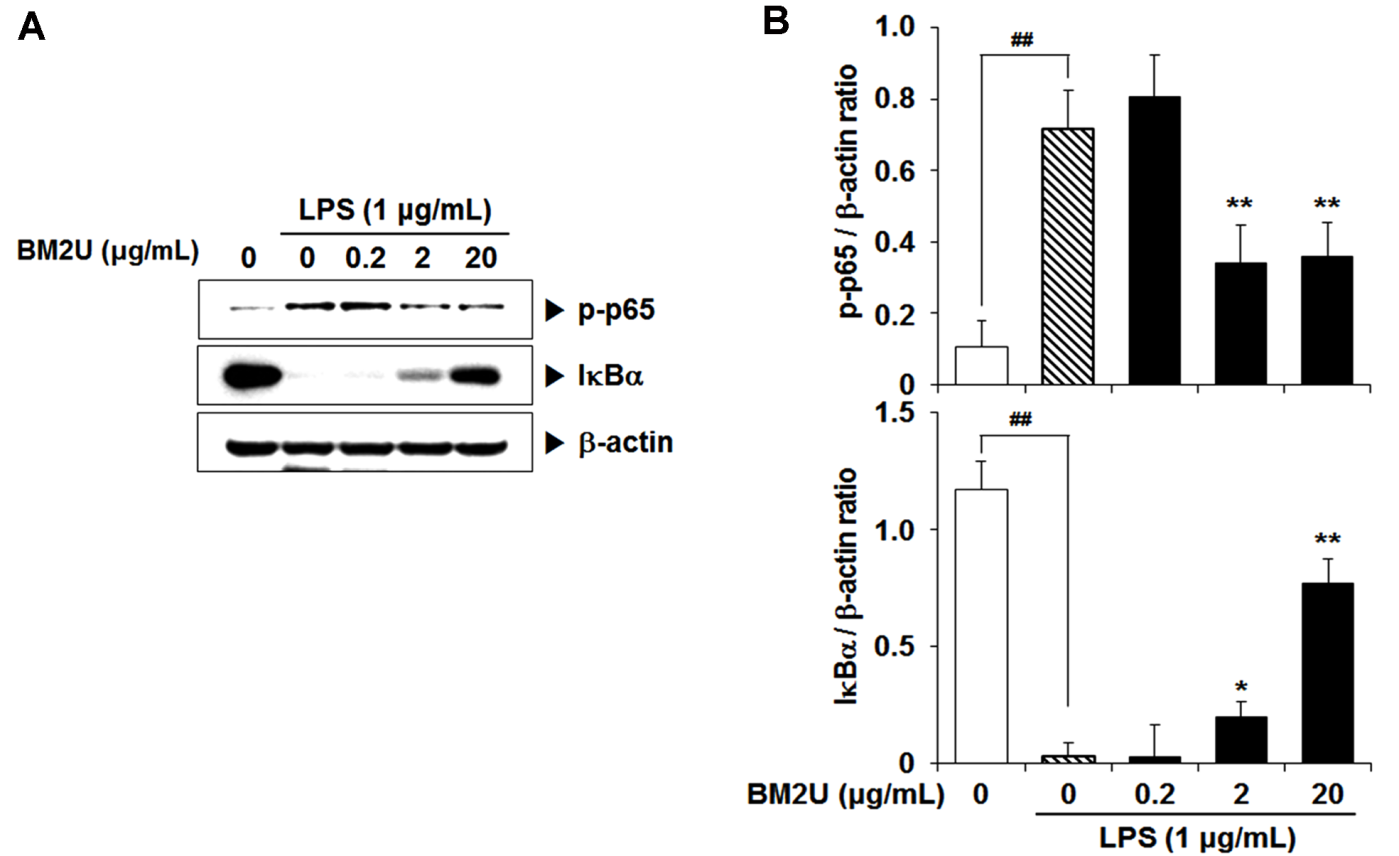
Effect of BM2U on LPS-mediated NF-κB activation. RAW264.7 macrophage cells were challenged with LPS (1 μg/ml) in the presence or absence of BM2U (0.2-20 μg/ml) for 15 min, and the expression levels of p-p65 and IκBα were quantified by immunoblot analyses. Values represent the relative ratio to β-actin as mean ± SEM (*n* = 3, per group). ##*p* < 0.01 vs. the untreated control; *p < 0.05 or **p < 0.01 vs. the LPS-only group. BM2U, an aqueous extract of *D. actinosclerus* BM2^T^ (GenBank: KT448814).

As shown in Fig. 2, BM2U treatment significantly inhibited LPS-mediated upregulation of TNF-α, IL-1β, and IL-6 mRNA, as well as COX-2 and iNOS. Considering the involvement of the transcription factor NF-κB in the upregulation of those proinflammatory cytokines, and the induction of iNOS and COX-2, we determined the effect of BM2U on LPS-mediated NF-κB activation. NF-κB activation has been associated with increased p65 phosphorylation (p-p65) and downregulation of the NF-κB inhibitory protein, IκBα. As depicted in Fig. 3, the LPS challenge on RAW264.7 macrophages led to significantly increased expression of p-p65, and decreased expression of IκBα. However, at 2 and 20 μg/ml, BM2U significantly reversed the increased expression of p-p65, and decreased expression of IκBα. These results suggest that LPS-induced NF-κB activation can be attenuated, at least in part, in the presence of BM2U. This suppressive effect of BM2U on NF-κB activation may result in the suppression of proinflammatory cytokines, and induction of COX-2 and iNOS.

### Effect of BM2U on LPS-Mediated Activation of MAPKs

**Fig. 4:**
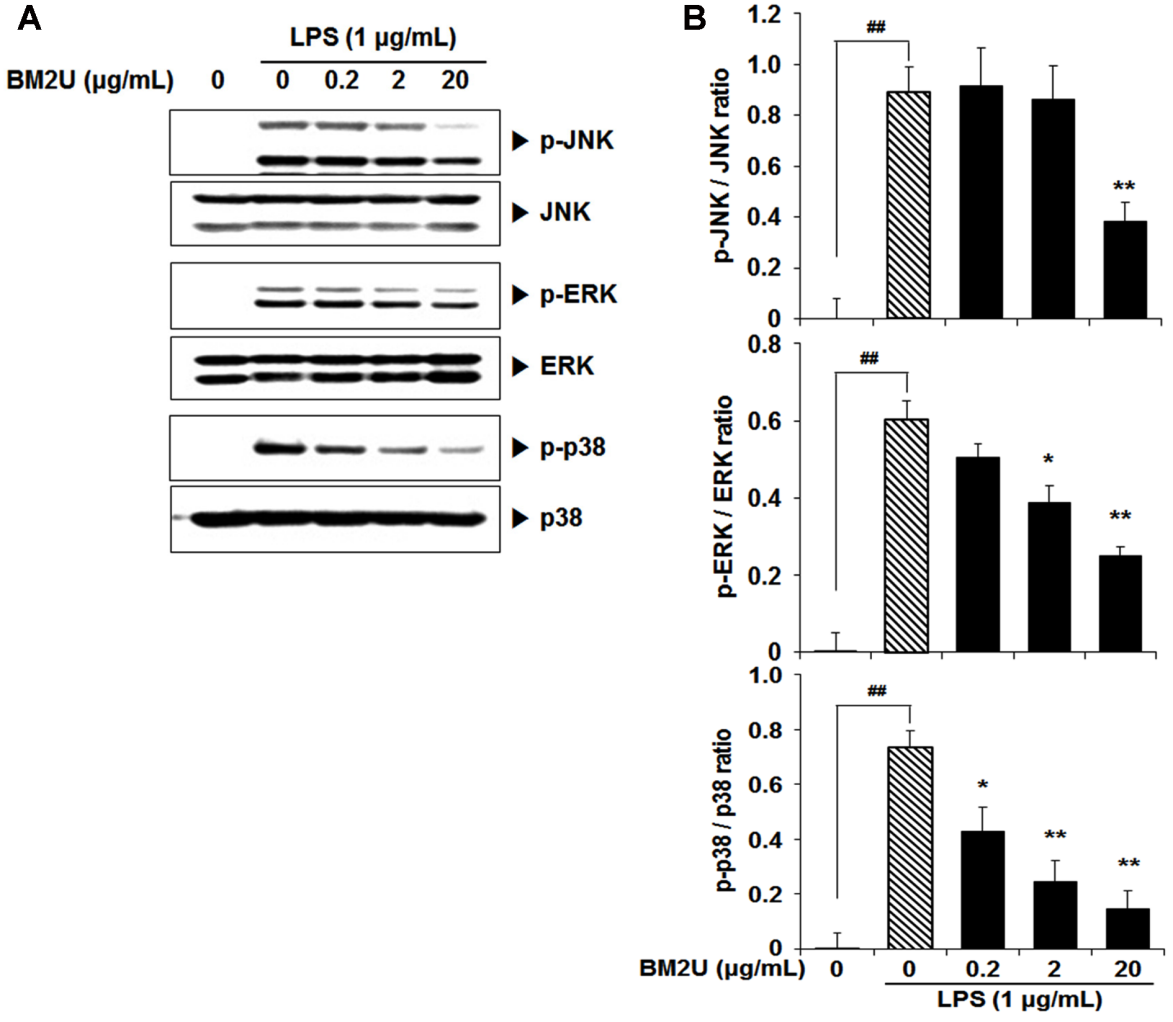
Effect of BM2U on LPS-mediated JNK, ERK, and p38 MAPK activation. RAW264.7 macrophage cells were challenged with LPS (1 μg/ml) in the presence or absence of BM2U (0.2-20 μg/ml) for 15 min. The phosphorylated JNK, ERK, and p38 MAPK levels, and their total form, were quantified by immunoblot analyses. Values represent the relative ratio to β-actin as mean ± SEM (*n* = 3, per group). ##*p* < 0.01, vs. the untreated control; *p < 0.05 or **p < 0.01 vs. the LPS-only group. BM2U, an aqueous extract of *D. actinosclerus* BM2^T^ (GenBank: KT448814).

MAPKs play a critical role in the expression of various cytokines and chemokines; thus, LPS-induced MAPKs inhibition can act as another restriction point for inflammatory responses. In addition, MAPKs are involved in the activation of the NF-κB signaling pathway [7]. As depicted in Fig. 4, the LPS challenge on RAW264.7 macrophages caused significant activation of ERK, JNK, and p38 MAPK (*p* < 0.05). However, these activations were significantly suppressed in the presence of BM2U. Similar to NF-κB inhibition (Fig. 3), BM2U-induced suppression of these 3 MAPKs may further suppress LPS-induced inflammatory responses.

### Effect of BM2U on HO-1 Expression

**Fig. 5:**
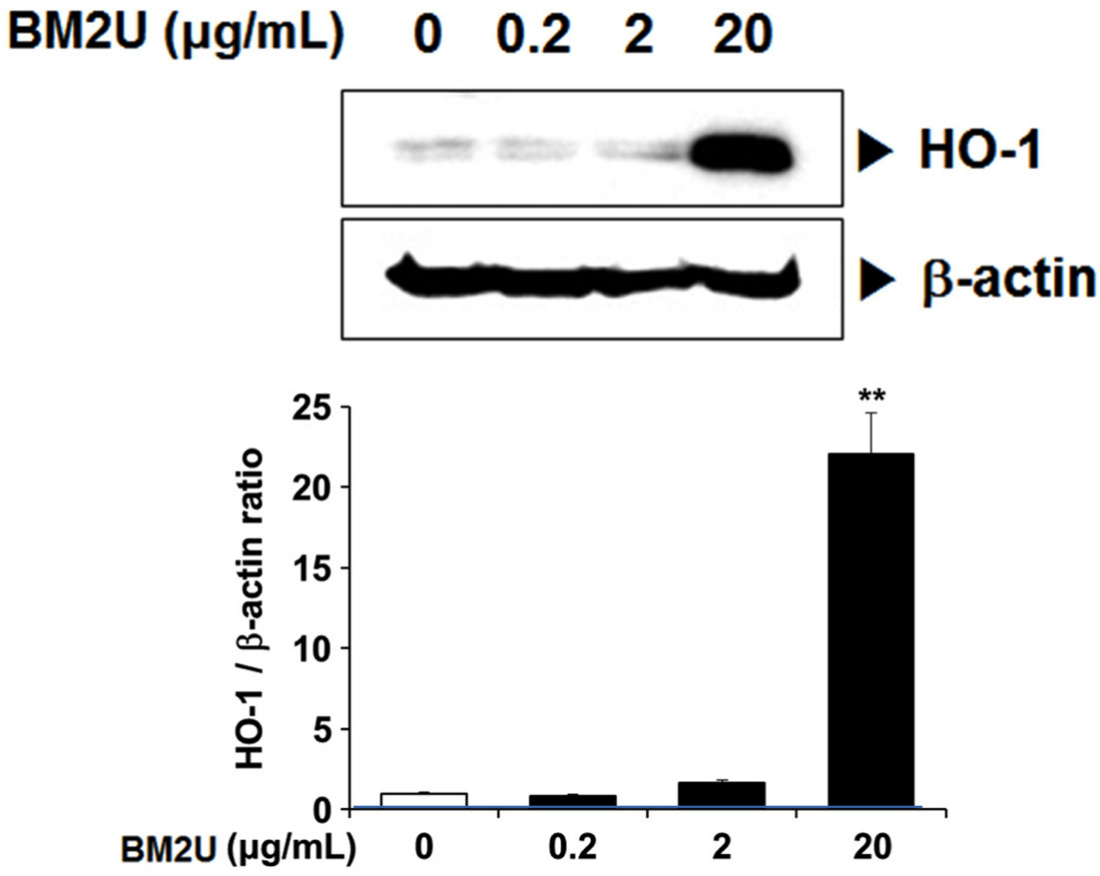
Effect of BM2U on the induction of HO-1. RAW264.7 macrophages were treated with BM2U (0.2-20 μg/ml) for 24 h. HO-1 levels were then quantified by immunoblot analyses. Values represent the relative ratio to β-actin as mean ± SEM (*n* = 3, per group). **p < 0.01 vs. the untreated control. BM2U, an aqueous extract of *D. actinosclerus* BM2^T^ (GenBank: KT448814). HO-1, heme oxygenase-1.

Apart from the NF-κB signaling pathway, the nuclear factor erythroid 2-related factor 2 (Nrf2) signaling pathway contributes to repressing oxidative stress and exerts its anti-inflammatory effects via the induction of HO-1 expression, because HO-1 attenuates inflammatory responses, and drives the phenotypic shift to M2 macrophages [16]. Hence, we examined if BM2U could induce HO-1 expression in RAW264.7 macrophage cells, increasing the anti-inflammatory potential of BM2U (Fig. 5). A significant induction of HO-1 was observed at 20 μg/ml BM2U (*p <* 0.05) and this HO-1 inducible activity of BM2U suggests further inflammatory modulation potential at higher but non-toxic concentrations.

## Discussion

In this study, we have demonstrated the anti-inflammatory potential of BM2U, an aqueous extract of BM2^T^ strain, on LPS-challenged RAW264.7 macrophages. BM2U treatment suppressed LPS-mediated NF-κB activation, resulting in the suppression of proinflammatory cytokines (TNF-α, IL-1β, and IL-6), and iNOS and COX-2 proteins involved in inflammatory mediator production. Three MAPKs (ERK, JNK, and p38 MAPK), which are major contributors to the progression of inflammation, were significantly inhibited by BM2U treatment (*p <* 0.05). In addition to its antioxidant capacity, BM2U induced HO-1 expression, known to contribute to antioxidant defense and anti-inflammatory activity.

Inflammation caused by infection or non-infectious etiologies, has a major impact on health and quality of life, and may trigger many chronic diseases [5]. There is a strong public need for natural and less expensive, but more effective anti-inflammatory drugs with less adverse effects [22]. In addition to classical synthetic drugs, plants and other natural products are the best sources of anti-inflammatory drugs [22]. Microbial-based therapies, such as beneficial bacterial transplants and bacterial intake as probiotics, have demonstrated the ability to reduce both dysbiotic environments and inflammatory mediator production; thus, inducing remission, specifically in ulcerative colitis [23]. To obtain novel applicability for BM2^T^ on a biological system, we investigated the anti-inflammatory potential of BM2U in LPS-challenged RAW264.7 macrophages. Inflammatory cytokines, chemokines, and interferons are major triggers of inflammatory responses, and these mediators are produced by interactions with pathogen and membrane receptors [3, 7]. Many studies have shown that the increase in inflammatory factors, such as NO, PGE2, iNOS, COX-2, TNF-α, and IL-6, in LPS-induced RAW264.7 cells, is mediated through the MAPK pathways [24-26]. BM2U treatment reduced mRNA expression levels of IL-1β, IL-6, and TNF-α (Fig. 2). The decreased production of these cytokines in the LPS-challenged RAW264.7 macrophages appeared to be associated with the suppression of NF-κB activation in the presence of BM2U (Fig. 3). Since NF-κB activation *via* proinflammatory cytokines is also possible, it is unclear whether the decreased cytokine expression shown in Fig. 2 is strictly associated with NF-κB inhibition. BM2U treatment attenuated the induction of iNOS and COX-2 (Fig. 2), which is known to produce the inflammatory mediators NO and PGE2, respectively. These results suggest that BM2U may attenuate the expansion of inflammation *via* inhibiting inflammatory mediator production. M1 macrophages are proinflammatory and responsible for inflammatory signaling, while M2 macrophages are anti-inflammatory and contribute to resolving inflammatory processes resulting in tissue healing [27]. We observed that BM2U could down-regulate the mRNA (IL-6, IL-1β, and TNF-α) and protein (iNOS and COX-2) levels of LPS-induced M1 biomarkers (IL-6, IL-1β, TNF-α, iNOS, and COX-2). Several studies also suggest that Nrf2/HO-1-mediated induction of antioxidant gene expression can reduce the phenotypic transition into the M1 macrophage phenotype [28, 29]. As revealed by DPPH and ORAC scavenging assays (Fig. 1), BM2U possesses antioxidant activities. It is not clear if this antioxidant activity is associated with antioxidant enzymes and/or non-enzymatic antioxidants, such as carotenoids. Clear identification of BM2U compounds involved in radical scavenging will be valuable in finding a new antioxidant source. Antioxidants and antioxidative proteins regulated by Nrf2 and the repressor factor Bach-1 (BTB and CNC homology 1) play a significant role in the suppression of inflammation [17, 30, 31]. In addition to Nrf2, transcription factors, such as AP-1 (activator protein 1), ATF1 (activating transcriptional factor 1), and NF-κB, are involved in the activation of HO-1 gene expression with complex mechanisms [17]. The ability of BM2U to induce HO-1, as well as its antioxidant capacities, may be interesting because in biological systems increased HO-1 synthesis usually occurs as a general response to stress [32]. It is possible that, as shown in traditional fermented foods [33], bacterial components involved in radioresistance of *D. actinosclerus* BM2^T^ may behave as Nrf2 co-factors resulting in the activation of Nrf2. However, the exact mechanism of BM2U-mediated HO-1 induction and its involvement in the suppression of the LPS-mediated inflammatory response requires further investigation. It should be mentioned that 0.2 and 2 μg/mL BM2U potently inhibited proinflammatory proteins (Fig. 2) in the absence of HO-1 induction (Fig. 5). These results strongly suggest that BM2U attenuates LPS-mediated inflammatory responses via suppression of NF-κB and activation of MAPKs. However, there was more potent inhibition of COX-2, p-JNK, and IκBα at 20 μg/ml BM2U where significant induction of HO-1 was observed. Therefore, BM2U may elicit anti-inflammatory effects via the HO-1-dependent and HO-1-independent pathways. The exact contribution of HO-1 induced by 20 μg/ml BM2U treatment should be investigated further in future studies.

In conclusion, BM2U, the aqueous extract of *Deinococcus actinosclerus* BM2^T^, possesses antioxidant capacity, and suppresses LPS-mediated increases in proinflammatory cytokine (TNF-α, IL-1β, and IL-6) mRNA expression, and iNOS and COX-2 induction, in RAW264.7 macrophage cells. The anti-inflammatory potential of BM2U can be associated with its inhibitory effects on NF-κB activation, and 3 MAPKs (p-ERK, p-JNK, and p-p38). Additionally, BM2U induces HO-1 upregulation. The anti-inflammatory properties of BM2U can provide novel insights into developing new strategies for treating chronic inflammation, such as in sepsis and radiation injury.

## Figures and Tables

**Table 1:** Real-time PCR Primer sequences.

Gene name	Primer sequences
Interleukin-1β (IL-1β)	5’- GCAACTGTTCCT GAACTCAACT-3’ (sense)
	5’- ATCTTTTGGGG TCCGTCAACT-3’ (anti-sense)
Interleukin-6 (IL-6)	5’- TGGAGTCACAGAAGGAGTGGCTAAG-3’ (sense)
	5’- TCTGACCACAGTGAGGAATGT CCAC-3’ (antisense)
Tumor necrosis factor-α (TNF-α)	5’-CCCTCACACTCAGAT CATCTTCT-3’ (sense)
	5’-GCTACGACGTGGGCTACAG -3’ (antisense)
β-actin	5’-TGTCCACCTTCCAGCAGATGT-3’ (sense)
	5’-AGCTCAGTAACAGTCCGCCTAGA-3’ (antisense)
